# Detection of pro angiogenic and inflammatory biomarkers in patients with CKD

**DOI:** 10.1038/s41598-021-87710-0

**Published:** 2021-04-22

**Authors:** Diana Jalal, Bridget Sanford, Brandon Renner, Patrick Ten Eyck, Jennifer Laskowski, James Cooper, Mingyao Sun, Yousef Zakharia, Douglas Spitz, Ayotunde Dokun, Massimo Attanasio, Kenneth Jones, Joshua M. Thurman

**Affiliations:** 1 Division of Nephrology, Department of Internal Medicine, Carver College of Medicine, University of Iowa, Iowa City, IA USA; 2 Carver College of Medicine, University of Iowa, Iowa City, IA USA; 3 Iowa City VA Medical Center, Iowa City, IA USA; 4 Department of Pediatrics, University of Colorado Anschutz Medical Center, Aurora, CO USA; 5 Renal Division, University of Colorado Anschutz Medical Center, Aurora, CO 80045 USA; 6 Institute for Clinical and Translational Science, University of Iowa, Iowa City, IA USA; 7 Department of Internal Medicine (Hematology and Oncology), Holden Comprehensive Cancer Center, University of Iowa, Iowa City, IA USA; 8 Division of Endocrinology and Metabolism, Department Internal Medicine, Carver College of Medicine, University of Iowa, Iowa City, IA USA; 9 Free Radical and Radiation Biology Program, University of Iowa, Iowa City, IA USA; 10 Harold Hamm Diabetes Center, School of Medicine, University of Oklahoma, Oklahoma City, OK USA

**Keywords:** Bioinformatics, Kidney diseases, Cardiovascular biology

## Abstract

Cardiovascular disease (CVD) is the most common cause of death in patients with native and post-transplant chronic kidney disease (CKD). To identify new biomarkers of vascular injury and inflammation, we analyzed the proteome of plasma and circulating extracellular vesicles (EVs) in native and post-transplant CKD patients utilizing an aptamer-based assay. Proteins of angiogenesis were significantly higher in native and post-transplant CKD patients versus healthy controls. Ingenuity pathway analysis (IPA) indicated Ephrin receptor signaling, serine biosynthesis, and transforming growth factor-β as the top pathways activated in both CKD groups. Pro-inflammatory proteins were significantly higher only in the EVs of native CKD patients. IPA indicated acute phase response signaling, insulin-like growth factor-1, tumor necrosis factor-α, and interleukin-6 pathway activation. These data indicate that pathways of angiogenesis and inflammation are activated in CKD patients’ plasma and EVs, respectively. The pathways common in both native and post-transplant CKD may signal similar mechanisms of CVD.

## Introduction

Approximately one in 10 individuals has chronic kidney disease (CKD) rendering CKD one of the most common diseases worldwide^1^. CKD is associated with a high burden of morbidity in the form of end stage kidney disease (ESKD) requiring dialysis or transplantation^2^. Furthermore, patients with CKD are at significantly increased risk of death from cardiovascular disease (CVD)^3,4^. In fact, CKD patients are 20 times more likely to die from CVD than they are of reaching ESKD^4^. The association between CKD and CVD is only partially explained by the high prevalence of traditional CVD risk factors (such as diabetes and hypertension) in CKD^5,6^. Multiple studies have shown that CKD, itself, is an independent and powerful predictor of cardiovascular events and mortality^3,7–10^. Hence, it has been postulated that “non-traditional” risk factors, such as inflammation, oxidative stress, and endothelial dysfunction contribute to the increased risk of CVD and mortality in CKD^11–15^. However, the underlying pathophysiology of CVD in CKD remains incompletely understood.

After kidney transplantation, the risk of CVD is significantly reduced, and yet CVD remains the most common cause of death and graft loss post-kidney transplantation^16–18^. Of interest, several studies have confirmed the limited predictive value of the traditional CVD risk factors in post-kidney transplantation patients^19–21^. Importantly, reduced kidney function is an independent predictor of CVD in post-kidney transplant patients, much like it is prior to transplantation^22,23^. Collectively, the literature suggests that the persistent risk of CVD post-kidney transplantation may be due to the same “non-traditional” CVD risk factors that affect patients with native kidney CKD. Endothelial dysfunction has been reported in patients with CKD post-kidney transplantation by our group and others^14,24–26^. Furthermore, in post-kidney transplantation patients, endothelial dysfunction has been shown to correlate with biomarkers of inflammation^26^. As such, endothelial dysfunction may represent a common underlying mechanism of CVD in CKD patients, pre- and post-kidney transplantation.

In order to develop new therapies for the prevention of CVD in CKD patients, biomarkers are needed that can accurately predict or reflect vascular injury in this population. A challenge in searching for new plasma biomarkers is that abundant plasma proteins, such as albumin, make it difficult to detect low abundance proteins by standard proteomic methods. To detect low abundance plasma proteins one can use assays designed to detect specific analytes (e.g. enzyme linked immunosorbent assays; ELISAs) or array-based assays. One can also use various methods to isolate or enrich specific fractions of the plasma proteome. We recently used both of these approaches to analyze plasma samples from CKD patients^14^. We isolated EVs by ultracentrifugation, and then measured the EV proteome with a SOMAscan assay. The SOMAscan assay uses aptamers to quantify > 1300 different proteins, and it can detect the proteins down to the femtomolar range. EVs are sub-micrometer-sized membrane bound particles that are actively shed from cells in response to activation, injury, and apoptosis^27,28^. Given the small quantity of protein contained in the EV fraction of plasma, the high sensitivity of the SOMAscan provides a useful method for analyzing the EV proteome. One surprising result of our previous study was that several proteins that were expected to be soluble were enriched in the EV fraction. This indicates that some proteins may preferentially segregate with the soluble or with the EV fractions of plasma, and that there may be value in examining both fractions. Hence, in the current study, we hypothesized that CKD patients, pre- and post-kidney transplantation, may share a similar inflammatory profile, the biomarkers of which correlate with endothelial dysfunction. We sought to characterize the inflammatory profile of the proteome of patients with CKD of the native kidneys and of the post-transplant kidneys compared to healthy controls utilizing the SOMAscan assay. Considering the potential differences between the plasma and EV fractions, we evaluated the proteome of both.

## Methods

### Patient characteristics

This is a sub-analysis of a previously published pilot study^14^. The ‘parent’ study included 30 healthy subjects, 30 patients with stage 3 or 4 CKD, and 30 patients post kidney transplantation with stage 3 or 4 CKD. The detailed recruitment procedures for the ‘parent’ study have been published^14^. Briefly, healthy subjects were recruited by public advertisement; the only exclusion criteria being pregnancy or breastfeeding. Patients with CKD were recruited from the CKD clinic and kidney transplant recipients were recruited from the transplant clinic (both clinics are at the University of Colorado Hospital). Individuals with CKD and kidney transplant recipients were considered eligible for participation if they were at least 18 years of age, had stage 3 or 4 CKD with a CKD-EPI estimated glomerular filtration equation (eGFR) of 20–59 mL/min/1.73m^2^^29^, and were able to give informed consent. In addition, kidney transplant recipients were required to be taking maintenance immunosuppression consisting of tacrolimus, mycophenolate mofetil, and corticosteroids. CKD patients and kidney transplant recipients were excluded if they were pregnant or breastfeeding, had uncontrolled hypertension, body mass index (BMI) ≥ 40 kg/m^2^, life expectancy < 1 year (defined as patient on hospice, terminal malignancy, history of significant liver disease, or significant congestive heart failure [ejection fraction < 20%]), hospitalizations within the last 3 months, or active infection on antibiotic therapy. For individuals with CKD of their native kidneys, history of immunosuppressive therapy in the last year was an additional exclusion criterion. The study was approved by the Colorado Multiple Institutional Review Board and all the study procedures were conducted in accordance with the federal human subjects regulations. The study procedures were conducted during 1 visit at the Clinical and Translational Research Center (CTRC) at the University of Colorado Anschutz Medical Campus after patients provided informed consent. During this visit, the study team collected the pre-determined clinical variables including blood pressure and body weight and height, obtained a blood draw for clinical labs and EDTA plasma (the latter was aliquoted and stored at − 80 °C). In order to validate our findings, we pursued an additional cohort of patients with CKD stage 3b and 4. We randomly selected samples from subjects who had enrolled to participate in 2 currently ongoing clinical trials. The first (NCT03223883) is recruiting patients with stage 3b and 4 CKD to evaluate whether curcumin improves vascular and cognitive function in patients in patients with CKD. In addition to stage 3b and 4 CKD, subjects must be 45–75 years old and have BMI < 35 kg/m^2^ and able to give informed consent. Exclusion criteria include: consuming a diet rich in curcumin or taking curcumin supplements in the past 12 months, pregnant, breastfeeding, or unwilling to use adequate birth control, uncontrolled hypertension, life expectancy < 1 year (defined as patient on hospice, terminal malignancy, history of significant liver disease, or significant congestive heart failure [ejection fraction < 20%]), hospitalization within the last 3 months, active infection or antibiotic therapy, or received immunosuppressive therapy within the last year. The second trial (NCT03597568) is designed to evaluate if resveratrol supplementation improves vascular function in patients with stage 3 CKD and diabetes mellitus (DM). The inclusion criteria are age 45–80 years, stage 3 CKD, diagnosis of type 2 DM, use of angiotensin converting enzyme inhibitor or angiotensin II receptor blocker for > 3 month prior to the study, and the ability to give informed consent. The exclusion criteria include: > 2 glasses/day red wine and/or taking resveratrol or vitamin C supplement in the past 12 months, BMI > 40 kg/m^2^, pregnant, breastfeeding, or unwilling to use adequate birth control, uncontrolled hypertension, uncontrolled DM, life expectancy < 1 year (defined as patient on hospice, terminal malignancy, history of significant liver disease, or significant congestive heart failure [ejection fraction < 20%]), hospitalization within the last 3 months, active infection or antibiotic therapy, or received immunosuppressive therapy within the last year. Of note, all the participating subjects had signed a consent agreeing to the use of their samples in future research and only samples from the baseline visit were utilized.

### Clinical variables

Race/ethnicity were based on patient self-reporting. DM status was defined either as history of DM according (obtained from the medical record), current treatment with oral hypoglycemic agents or with insulin, or fasting glucose ≥ 126 mg/dL. BMI was calculated as kg/m^2^. Blood pressure was obtained via an automated cuff after 10 min of rest at the beginning of the study visit. Serum creatinine and albumin to creatinine ratio (ACR) were measured by the clinical lab. eGFR was calculated based on the CKD-EPI formula^29^ and urinary ACR was reported as mg/g.

### Extracellular vesicle isolation

Plasma EVs were isolated as previously described^30^. Briefly, EDTA plasma samples were and stored at − 80 °C until used. To separate the EVs, the samples were thawed in a 37 °C water bath and were centrifuged at 400× *g* for 15 min at 4 °C. The supernatants were then collected and the volume recorded. Next, 250 µl of each sample was placed in a polyallomer tube. The plasma was centrifuged at 20,000× *g* for 2.5 h using a Beckman XL-80 ultracentrifuge with a sw55ti rotor. Braking was applied at the end of the centrifugation. EV buffer (Hank’s buffered saline solution containing 20 mM HEPES and 5 mM glucose) was used to re-suspend the pellets to a volume of 100 µl (or 40% of the initial plasma volume) by pipetting up and down six times^31^. To characterize the EVs, we performed Western blot analysis on a protein lysate generated from the EV fraction of 3 mL of plasma from a CKD patient^31^. Protein samples were separated by electrophoresis on 10% Criterion TGX gels (Biorad, Hercules, CA) and transferred to Immulon P membrane (Millipore, Burlington, MA). The lysates were then probed using antibodies to CD63 (R&D Systems, Minneapolois, MN), CD81 (GenTex, Zeeland, MI), TSG101 (Novus Biologicals, Centennial, CO), and Calnexin (Novus Biologicals). A protein lysate generated from A549 cells (pulmonary epithelial cells) was used as a positive control for the Calnexin blot. The antibodies were detected with appropriate horse radish peroxidase (HRP)-conjugated secondary antibodies and electrochemiluminescence reagent (Pierce Biotechnology, Rockford, IL). Bands were seen in the blots probed for CD63, CD81, and TSG101 (Supplemental Fig. 1S). Only faint bands were seen in the blot probed for calnexin.

### Proteomic analysis

This was conducted on collected EDTA plasma and the isolated EVs. The isolated EVs were incubated in a 37 °C water bath for 15 min with occasional agitation then centrifuged at room temp for 5 min at 14,000 XG. Supernatants were subsequently collected, and protein concentrations were determined on a Nanodrop 2000 with secondary confirmation of concentration on select samples by BioRad (Hercules, CA) BCA protein concentration analysis. The Somalogic SOMAscan assay was then used to analyze the proteome in an EDTA plasma sample and an EV sample for each included subject at the University of Colorado Genomics and Microarray Core. The SOMAscan assay is a multiplexed proteomics assay that uses aptamers (single stranded DNA molecules) selected to bind specific protein targets. The bound proteins are then quantified. This assay is advantageous for analyzing complex samples because it has high very high sensitivity and wide dynamic range, and high abundance proteins in the sample (such as albumin) do not obscure the detection of low abundance proteins. The assay panel utilized detects > 1300 proteins. There are some limitations to this assay, however, including cross-reactivity of the aptamers with non-target proteins^32^.

Level of Ephrin B2 in the plasma was determined via enzyme-linked immunosorbent assay (ELISA) by a commercially available kit (Biomatik, Wilmington, Del). Samples were run as described in the kit manual through the reagent B incubation step and subsequent washing. ELISA Amplification system (ELAST) [PerkinElmer LAS, Inc, Waltham, MA] was inserted to the reaction system according to the manufacturer's instructions. The ELAST streptavidin-HRP concentrate was diluted 1/750. Selected analytes were also measured using Meso Scale Discovery (MSD) assays (Rockville, MD). MSD uses electrochemiluminescence to detect proteins with high sensitivity. We used multiplex (VEGF-A and VEGF-D) and singleplex (CFD) assays according to the manufacturer’s instructions. Briefly, plates were coated and incubated overnight at 4 °C with continuous rotary shaking at 700 rpm. Plasma was either diluted two-fold and incubated for two hours (VEGF-A and VEGF-D) or 1000-fold and incubated for one hour (CFD). Analyte intensities were captured with the MESO QuickPlex SQ 120 instrument and analyzed with MSD Discovery Workbench, v4 software (Meso Scale Discovery).

### Statistical analysis

Descriptive statistics are reported by study group as N (%) for categorical variables and mean (SD) or median (IQR) for continuous variables. Fisher’s exact test was used to test for differences in categorical variables between the 3 different conditions, while the Kruskal–Wallis nonparametric test was used for continuous variables. In reporting individual protein expression, significance was set at a p value < 0.0002 in order to account for multiple comparisons. Ingenuity pathway analysis (IPA) was utilized as the bioinformatics approach for the proteomics analysis. IPA allows for the functional interpretation of data such as ours^33^ which we felt was important considering the pilot nature of our pursuit. First, we identified the proteins that were differentially expressed in the native and post-transplant CKD patients versus the healthy controls. Then, we ranked these proteins in order of magnitude and significance prior to conducting IPA analysis^34^. Proteins were ranked and included in the IPA if they were found to have differential expression > 1 or < 1 with a *p* value of < 0.05. Subsequently, we evaluated the potential correlation between urinary albumin/creatinine ratio (uACR) and the overlapping proteins (proteins found to be significantly different in native and post-transplant CKD versus healthy control). uACR was evaluated considering it is well known marker for increased CVD risk and mortality in addition to CKD progression^35,36^. We opted to evaluate these correlations in the combined group of subjects defined as native kidney CKD and post-kidney transplant CKD consistent with our hypothesis that CVD risk may share similar non-traditional pathways in both groups. Statistical analysis was carried out using SAS version 9.4 (SAS Institute, Cary NC).

## Results

### Clinical characteristics

The clinical characteristics for all the participants in the ‘parent’ study have been published elsewhere^14^. We identified 16 healthy controls, 16 CKD patients (stage 3 or 4), and 16 kidney transplant recipients with CKD who had residual EDTA plasma samples at a volume adequate for the analysis. The clinical characteristics of the included subjects are shown in Table 1. The healthy controls were younger, mostly women, had lower BMI and blood pressure and higher eGFR (Table 1A). The clinical characteristics for the validation cohort are shown in Table 1B.

**Table 1:** Clinical characteristics of participants.

	CKD	Post-transplant CKD	Healthy control	*p* value (ANOVA)
	Stage 3 and 4	Stage 3 and 4		
	(n = 16)	(n = 16)	(n = 16)	
*A: Original cohort*				
Age (years)	58 ± 17	52 ± 14	36 ± 14	0.0017
Gender (female %)	31%	50%	75%	0.05
Race (Caucasian %)	81%	100%	88%	0.45
History of DM (%)	47%	7%	0%	0.001
BMI (kg/m^2^)	29.0 ± 5.3	25.9 ± 4.3	25.2 ± 5.8	0.1
SBP (mmHg)	131 ± 14	135 ± 14	115 ± 12	0.0006
DBP (mmHg)	80 ± 8	83 ± 10	71 ± 8	0.002
CKD-EPI eGFR (mL/min/1.73m^2^)	41 ± 9	49 ± 10	100 ± 17	< 0.0001
ACR (mg/g)	6 ± 17	1 ± 3	0.1 ± 0.1	0.02
ACEi/ARB (%)	73	30	0	< 0.0001
B: Validation Cohort* (n = 30)				
Age (years)	64 ± 9			
Gender (female %)	32%			
Race (Caucasian %)	92%			
History of DM (%)	39%			
BMI (kg/m^2^)	29.8 ± 4.5			
SBP (mmHg)	137 ± 15			
DBP (mmHg)	76 ± 9			
CKD-EPI eGFR (mL/min/1.73m^2^)	36 ± 11			
ACR (mg/g)	7.5 ± 17			
ACEi/ARB (%)	64			

Values are expressed as means ± standard deviation or % = percent of patients; DM = diabetes mellitus; BMI = body mass index; SBP = systolic blood pressure; DBP = diastolic blood pressure; CKD-EPI eGFR = CKD-EPI estimated glomerular filtration rate; ACR = urinary albumin/creatinine ratio; ACEi: angiotensin converting enzyme inhibitor; ARB: angiotensin receptor II blocker.

*CKD only.

Nine subjects in each group had adequate sample remaining for EV analysis. The clinical characteristics for the subjects with adequate sample for the EV analysis are shown in Supplementary Table 1S.

### Proteins altered in plasma of native kidney CKD patients

**Figure 1:**
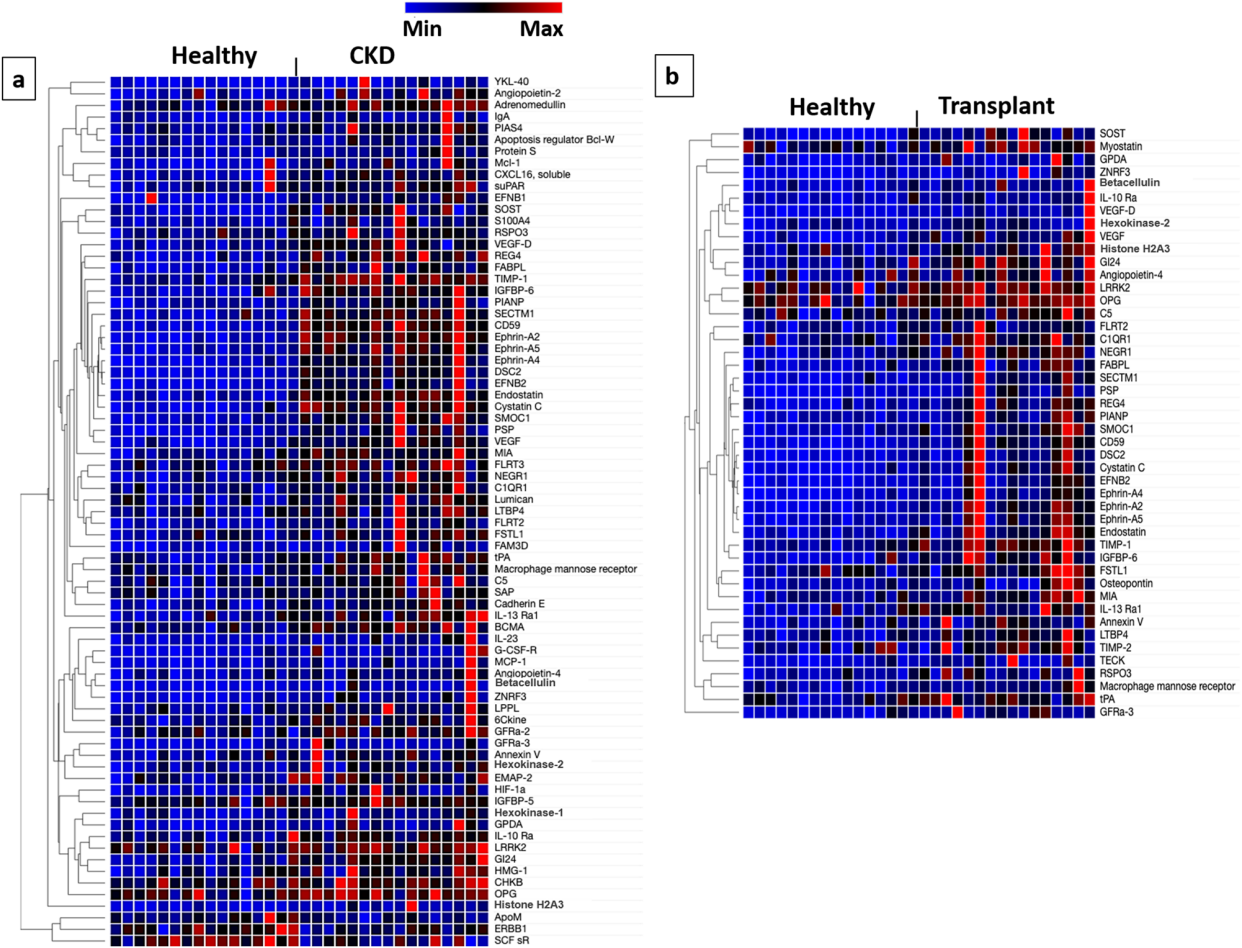
Plasma proteins differing between healthy controls and CKD. (**a**) illustrates the proteins found to be significantly different in the plasma of the stage 3 or 4 CKD patients as compared to the healthy controls. (**b**) shows the proteins found to be significantly different between the post-transplant patients with stage 3 or 4 CKD as compared to the healthy controls. Considering the large number of proteins detected by this assay, we only included those with any differences between the CKD subjects and the post-transplant subjects as compared to the healthy controls. The heat map was created via Morpheus, https://software.broadinstitute.org/morpheus. *ABV:* CKD: chronic kidney disease, Transplant: post-transplant CKD, YKL-40: also chitinase-3-like protein 1 (CHI3L1), IgA: immunoglobulin A, Mcl-1: Myeloid Cell Leukemia Sequence 1, CXCL16: Chemokine (C-X-C motif) ligand 16, suPAR: soluble urokinase plasminogen activator receptor, EFN: ephrin, SOST: sclerostin, S100A4: S100 calcium-binding protein A4, RSPO3: R-Spondin 3, VEGF: vascular endothelial growth factor, REG4: regenerating islet-derived protein 4, FABPL: fatty acid-binding protein, liver-type, TIMP-1: tissue inhibitor of metalloproteinases, IGFBP: insulin-like growth factor-binding protein, PIANP: PILR Alpha Associated Neural Protein, SECTM1: secreted and transmembrane protein 1, Ephrin: Eph family receptor interacting proteins, DSC: desmocollin, SMOC1: secreted modular calcium-binding protein 1, PSP: phosphoserine phosphatase, MIA: melanoma inhibitory activity, FLRT: Fibronectin leucine-rich repeat transmembrane protein, NEGR: neuronal growth regulator, C1QR1: complement component C1q receptor, LTBP: latent-transforming growth factor beta-binding protein, FSTL: Follistatin-related protein, FAM3D: family with sequence similarity 3 member D, tPA: tissue plasminogen activator, C5: complement 5, SAP: SLAM-associated protein, IL-13Ra1: interleukin 13 Receptor Subunit α 1, BCMA: B cell maturation antigen, IL-23: interleukin-23, G-CSF-R: granulocyte colony-stimulating factor receptor, MCP-1: monocyte chemoattractant protein-1, ZNRF3: Zinc And Ring Finger 3, LPPL: lysophospholipase, 6Ckine/ CCL21: chemokine ligand 21, GFRa: GDNF family receptor α, EMAP: echinoderm microtubule-associated protein, HIF-1α: hypoxia-inducible factor- 1α, GPDA: glyceraldehyde-3-phosphate dehydrogenase, IL-10 Ra: interleukin-10 Rα, LRRK-2: dardarin, HMG: high mobility protein, CHKB: choline kinase beta, OPN: osteopontin, ApoM: apolipoprotein- M, ERBB1: erythroblastosis oncogene B-1, SCFsR: Stem Cell Factor Soluble Receptor, TECK/ CCL25: chemokine ligand 25.

The SOMAscan assay revealed significant differences in the plasma proteomes of the three study groups (Supplemental Fig. 2S). When evaluating individual proteins, several known to increase in patients with CKD, such cystatin C, tPA, and insulin-like growth factor binding protein (IGFBP)-6 were found to be increased in the CKD patients. While we anticipated considerable differences in biomarkers of inflammation, the most prominent differences between the healthy controls and the CKD patients were observed in proteins involved in angiogenesis. Figure 1a shows the heat map for all the proteins that differed between the CKD and healthy control groups. IPA identified the following top canonical pathways as significantly different for CKD versus healthy controls: ephrin receptor signaling, planar cell polarity (PCP), ephrin B signaling, serine biosynthesis, and role of macrophages, fibroblasts, and endothelial cells in rheumatoid arthritis. The following causal networks were identified as significantly activated: transforming growth factor (TGF)-β1, TGF-β2, activating transcription factor 4 (ATF4), and angiopoietin-1. In contrast, the Ras-related protein Ral-B (RalB) pathway was found by IPA to be inhibited in CKD patients.

Differentially expressed proteins included the ephrin ligand (EFN)- B2, Eph family receptor interacting proteins (ephrin) A2 and A5, and vascular endothelial growth factor (VEGF). Increased levels of additional proteins linked to angiogenesis were identified, including insulin-like growth factor-binding protein (IGFBP-6), secreted modular calcium-binding protein 1 (SMOC1), and endostatin. These data are summarized in Table 2.

**Table 2:** Proteins identified to be significantly different in plasma of CKD patients as compared to the healthy control group.

Target	CKD relative to healthy		Biological pathways
	CKD/healthy	*p* value	
PSP	2.4	< 0.0001	Cell proliferation and differentiation
Desmocollin 2	2.3	< 0.0001	Cell–cell junctions
Sclerostin	2.2	< 0.0001	Bone formation
Cystatin C	2.2	< 0.0001	Protein degradation
FABPL	2.1	< 0.0001	Fatty acid metabolism
REG4	2.1	< 0.0001	Cell proliferation, generation
tPA	1.9	< 0.0001	Fibrinolysis
IGFBP-6	1.9	< 0.0001	Cell proliferation, angiogenesis
CD59	1.9	< 0.0001	Complement-induced lysis, T cell activation
Ephrin-A4	1.8	< 0.0001	Angiogenesis
EFNB2	1.8	< 0.0001	Angiogenesis
SECTM1	1.8	< 0.0001	Immune response, inflammation
Ephrin-A2	1.8	< 0.0001	Angiogenesis
SMOC1	1.7	< 0.0001	Angiogenesis, fibrosis
Ephrin-A5	1.7	< 0.0001	Angiogenesis
VEGF-D	1.7	< 0.0001	Angiogenesis
PIANP	1.6	< 0.0001	Immune modulation
Endostatin	1.6	< 0.0001	Angiogenesis
MIA	1.6	< 0.0001	Cell proliferation, extracellular matrix
TIMP-1	1.5	< 0.0001	Extracellular matrix
VEGF-A	1.5	< 0.0001	Angiogenesis
NEGR-1	1.3	0.0001	Cell adhesion, axon sprouting

The data are shown ratiometrically for relative fluorescence units (RFU) for CKD/healthy control. CKD: chronic kidney disease, PSP: phosphoserine phosphatase, FABPL: fatty acid-binding protein, liver-type, REG4: regenerating islet-derived protein 4, tPA: tissue plasminogen activator, IGFBP-6: insulin-like growth factor-binding protein, EFN: ephrin, SECTM1: secreted and transmembrane protein 1, Ephrin: Eph family receptor interacting proteins, SMOC1: secreted modular calcium-binding protein 1, VEGF: vascular endothelial growth factor, PIANP: PILR Alpha Associated Neural Protein, MIA: melanoma inhibitory activity, TIMP-1: tissue inhibitor of metalloproteinases, NEGR: neuronal growth regulator.

### Proteins altered in plasma of post-kidney transplant CKD patients

In comparing the post-transplant CKD patients to the healthy controls, we identified a pattern similar to that observed in native kidney CKD. The heat map in Fig. 1b illustrates the proteins that differed most significantly between the post-transplant CKD and the healthy control groups. IPA identified the following top canonical pathways as significantly different: role of macrophages, fibroblasts, and endothelial cells in rheumatoid arthritis, serine biosynthesis, superpathway of serine and glycine biosynthesis I, IL-6 signaling, and ephrin receptor signaling. The following causal networks were identified as significantly activated: insulin growth factor (IGF)-1, phosphatase and tensin homolog (PTEN), TGF-β2, ATF4, four and a half LIM domains protein 2 (FHL2), and interleukin-1 receptor-like 1 (IL-1RL1). The following angiogenesis proteins were significantly higher in post-transplant CKD: IGFBP-6, VEGF-D, ephrin A2 and A4, EFN-B2, SMOC1, and endostatin. These findings are summarized in Table 3.

**Table 3:** Proteins identified to be significantly different for post-transplant CKD as compared to the healthy control group.

Target	Post-transplant CKD patients relative to healthy		Biological pathways
	Transplant/healthy	*p* value	
Cystatin C	1.9	< 0.0001	Protein degradation
IGFBP-6	1.9	0.0002	Cell proliferation, angiogenesis
REG4	1.8	< 0.0001	Cell proliferation, generation
CD59	1.6	< 0.0001	Complement-induced lysis, T cell activation
Ephrin-A4	1.6	0.0002	Angiogenesis
EFNB2	1.6	0.0001	Angiogenesis
SMOC1	1.5	< 0.0001	Angiogenesis, fibrosis
Ephrin-A2	1.5	< 0.0001	Angiogenesis
VEGF-D	1.5	< 0.0001	Angiogenesis
Endostatin	1.5	< 0.0001	Angiogenesis
TIMP-1	1.4	< 0.0001	Extracellular matrix
NEGR-1	1.3	< 0.0001	Cell adhesion, axonal sprouting

The data are shown ratiometrically for relative fluorescence units (RFU) for CKD/healthy control. CKD: chronic kidney disease, IGFBP-6: insulin-like growth factor-binding protein, VEGF: vascular endothelial growth factor, SECTM1: secreted and transmembrane protein 1, REG4: regenerating islet-derived protein 4, Ephrin: Eph family receptor interacting proteins, EFN: ephrin. *p* value was considered significant < 0.0002 for individuals proteins, TIMP-1: tissue inhibitor of metalloproteinases, NEGR: neuronal growth regulator.

### Overlapping proteins in the plasma of native kidney and post-kidney transplant CKD as compared to the healthy controls

**Figure 2:**
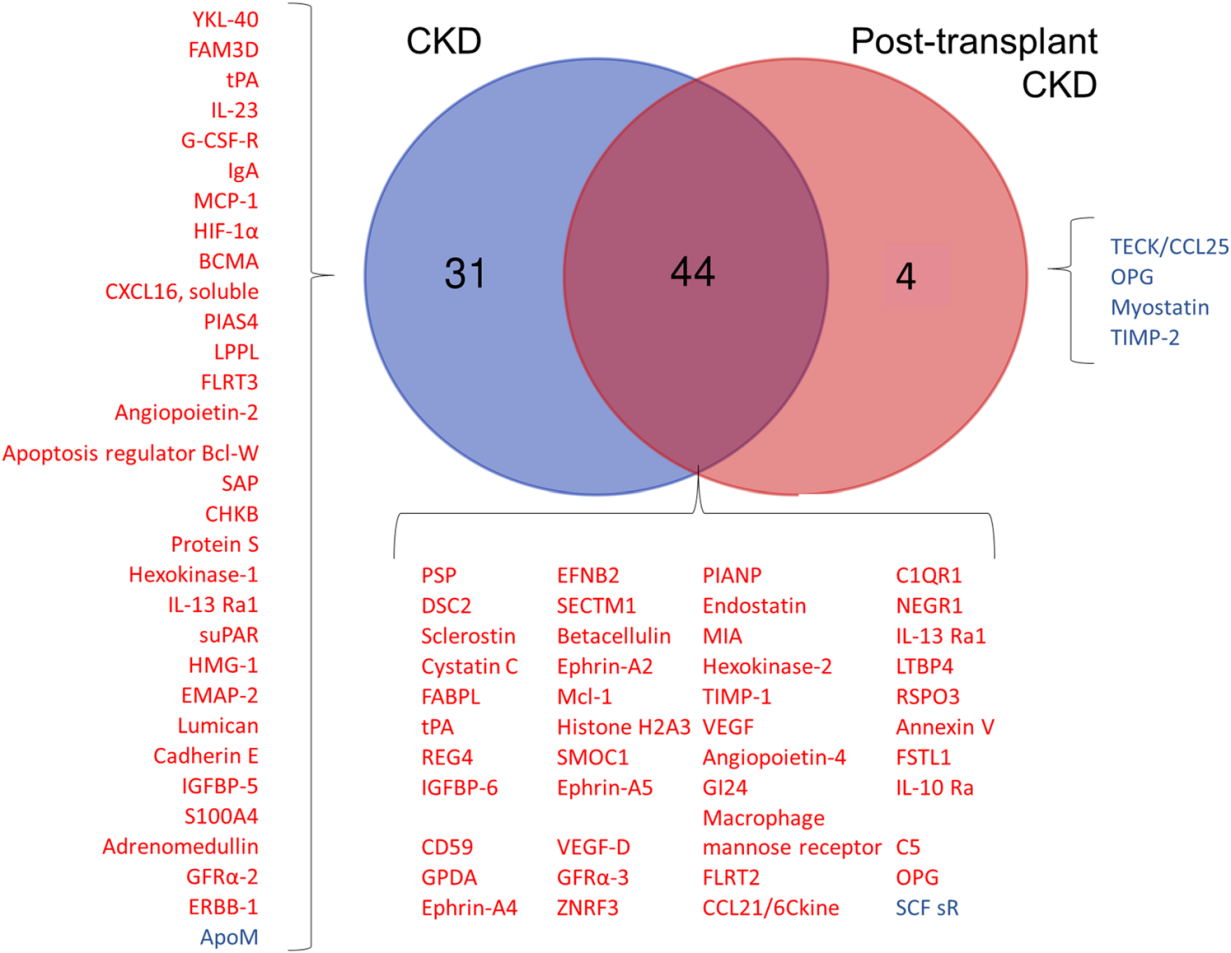
Proteins differing between healthy controls and patients with chronic kidney disease. A VENN diagram is shown for the proteins found to be significantly higher or lower in the plasma of CKD patients and the post-transplant CKD patients (each group compared to the plasma proteins of the healthy controls). Of those proteins, 44 were found to be shared as they were significantly higher in both the CKD patients and the post-transplant CKD patients. The red font was used to the proteins that were higher in CKD or in post-transplant CKD (vs. healthy). The blue font was used to denote the proteins that were lower in CKD or post-transplant CKD (vs. healthy controls). *ABV:* YKL-40: also chitinase-3-like protein 1 (CHI3L1), IgA: immunoglobulin A, Mcl-1: Myeloid Cell Leukemia Sequence 1, CXCL16: Chemokine (C-X-C motif) ligand 16, suPAR: soluble urokinase plasminogen activator receptor, EFN: ephrin, SOST: sclerostin, S100A4: S100 calcium-binding protein A4, RSPO3: R-Spondin 3, VEGF: vascular endothelial growth factor, REG4: regenerating islet-derived protein 4, FABPL: fatty acid-binding protein, liver-type, TIMP-1: tissue inhibitor of metalloproteinases, IGFBP: insulin-like growth factor-binding protein, PIANP: PILR Alpha Associated Neural Protein, SECTM1: secreted and transmembrane protein 1, Ephrin: Eph family receptor interacting proteins, DSC: desmocollin, SMOC1: secreted modular calcium-binding protein 1, PSP: phosphoserine phosphatase, MIA: melanoma inhibitory activity, FLRT: Fibronectin leucine-rich repeat transmembrane protein, NEGR: neuronal growth regulator, C1QR1: complement component C1q receptor, LTBP: latent-transforming growth factor beta-binding protein, FSTL: Follistatin-related protein, FAM3D: family with sequence similarity 3 member D, tPA: tissue plasminogen activator, C5: complement 5, SAP: SLAM-associated protein, IL-13Ra1: interleukin 13 Receptor Subunit α 1, BCMA: B cell maturation antigen, IL-23: interleukin-23, G-CSF-R: granulocyte colony-stimulating factor receptor, MCP-1: monocyte chemoattractant protein-1, ZNRF3: Zinc And Ring Finger 3, LPPL: lysophospholipase, 6Ckine/ CCL21: chemokine ligand 21, GFRa: GDNF family receptor α, EMAP: echinoderm microtubule-associated protein, HIF-1α: hypoxia-inducible factor- 1α, GPDA: glyceraldehyde-3-phosphate dehydrogenase, IL-10 Ra: interleukin-10 Rα, LRRK-2: dardarin, HMG: high mobility protein, CHKB: choline kinase beta, OPN: osteopontin, ApoM: apolipoprotein- M, ERBB1: erythroblastosis oncogene B-1, SCFsR: Stem Cell Factor Soluble Receptor, TECK/ CCL25: chemokine ligand 25.

After identifying the proteins that differed in the CKD patients versus the healthy controls and those that differed in the post-transplant CKD patients versus the healthy controls, we evaluated whether any of these proteins overlapped between the CKD patients and the post-transplant CKD patients. Of note, this analysis included all the proteins noted to be significantly different in comparing the CKD patients with the healthy controls or the post-transplant CKD patients with the healthy controls (i.e. all proteins with ratio of CKD/healthy or post-transplant/healthy > 1 or < 1). Figure 2 details all the significant proteins identified when comparing both the CKD and the post-transplant CKD groups to the healthy controls. We found 44 proteins that overlapped between the CKD and post-transplant CKD groups including several biomarkers of angiogenesis (IGFBP-6, ephrin A2 and A4, EFN- B2, SMOC1, VEGF, and endostatin).

uACR is a clinical indicator of vascular disease. In evaluating the 44 proteins that overlapped in between the native kidney CKD and post-kidney transplant CKD groups, the following were found to correlate significantly with uACR: desmocollin-2 (DSC-2), cystatin C, fatty acid-binding protein, liver-type (FABPL), regenerating islet-derived protein 4 (REG-4), IGFBP-6, CD59, ephrin-A2, A4, and A5, and EFN-B2. These data are shown in Table 5.

### Differences in the EV proteomic panel according to study group

**Figure 3:**
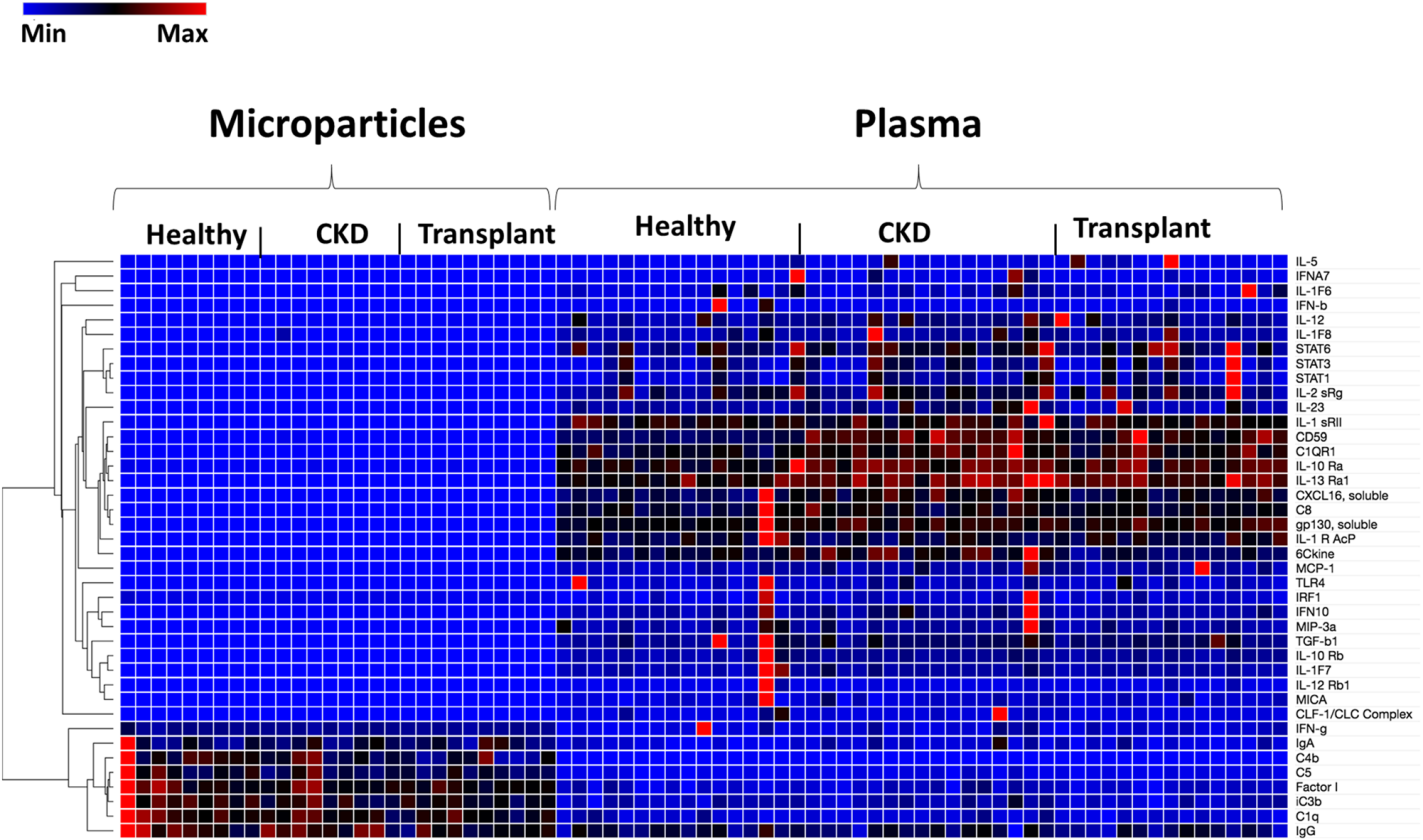
Comparison of the plasma and extracellular vesicle proteomes. The heatmap indicates that the proteins detected in the plasma differed significantly from the proteins identified by evaluating the circulating EVs. This observation was independent of the study group. The heat map was created via Morpheus, https://software.broadinstitute.org/morpheus. *ABV:* IL: interleukin, IFN: interferon, STAT: signal transducer and activator of transcription, C1QR1: complement component C1q receptor, CXCL: Chemokine (C-X-C motif) ligand, GP 130: glycoprotein 130, IL-1R AcP: IL-1R accessory protein, 6Ckine/CCL21: chemokine ligand 21, MCP: monocyte chemoattractant protein, TLR: toll-like receptor, IRF1: interferon regulatory factor-1 MIP 3A: macrophage inflammatory protein-3 (also CCL20), TGF-b1: transforming growth factor-β1, MICA:MHC Class I Polypeptide-Related Sequence A, CLF1/CLC complex: cytokine-like factor-1/cardiotrophin-like cytokine, IgA: immunoglobulin A, c4b: complement fragment 4b, c5: complement 5, Factor I: complement factor I, c3b: complement fragment 3b, IgG: immunoglobulin G.

Circulating EVs are derived from cells. They carry proteins from the parent cell, and plasma proteins can adhere to their surface^14,37^. We previously reported that the size and number of medium-sized (100–1000 nm) endothelial EVs was the same in samples from patients with CKD, transplant CKD, and healthy controls, but we did not analyze the protein content of the EVs^14^. The SOMAscan tissue assay was used to analyze the proteomes of the EVs from the three patient groups. We observed fewer significant differences between the circulating EV of the 3 study groups than we observed in the plasma samples. These data are shown in Supplemental Fig. 3S. The assays used for the plasma and for the EVs included 40 shared proteins. Comparison of the levels of these proteins between the plasma and EV samples demonstrates that the two types of samples contain distinct protein subsets (Fig. 3).

### Proteins altered in the circulating EVs of native kidney CKD patients

**Figure 4:**
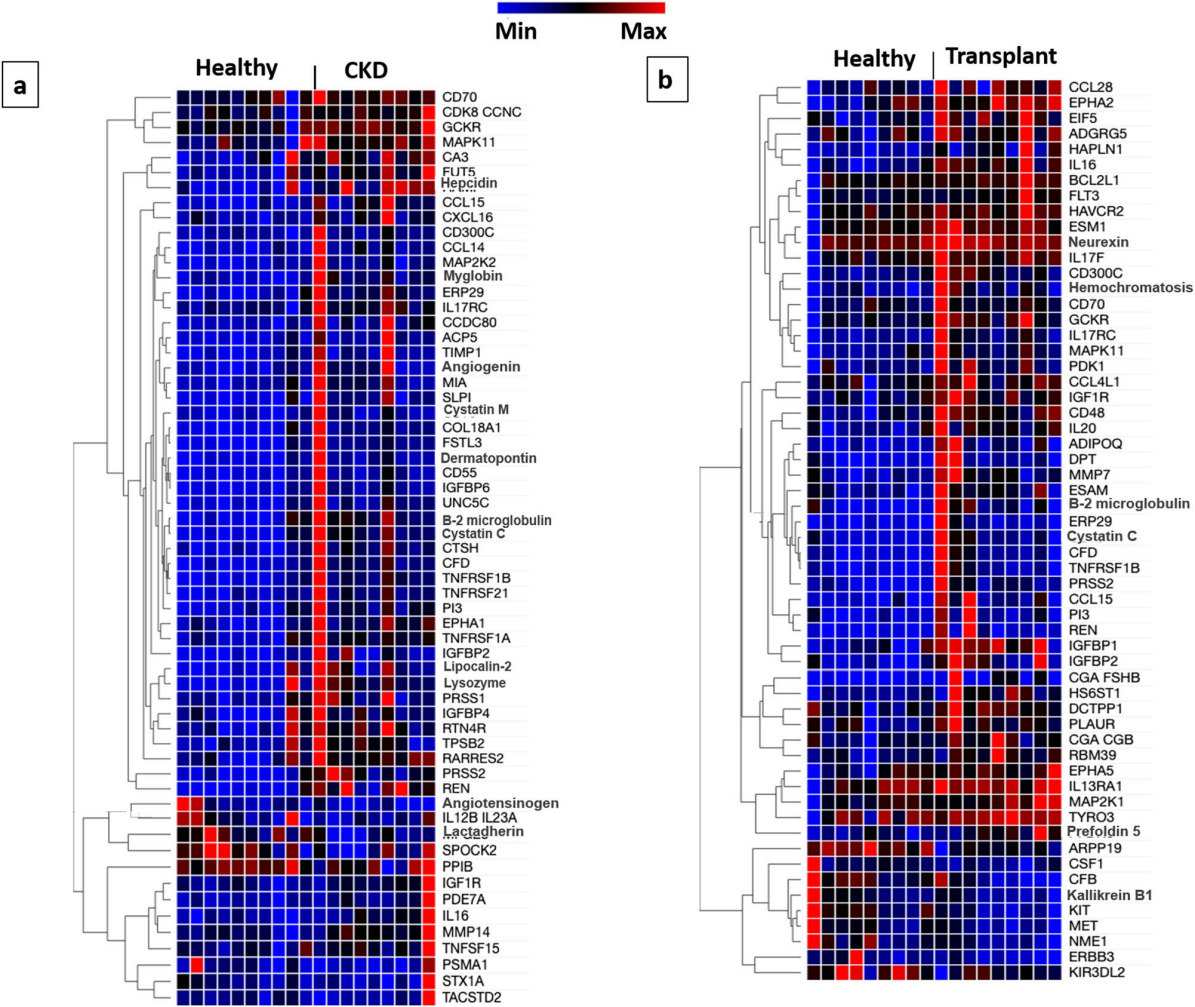
Extracellular vesicle proteins differing between healthy controls and CKD. (**a**) shows the proteins found to be significantly different in the EVs of the patients with stage 3 or 4 CKD as compared to the healthy controls. (**b**) illustrates the proteins found to be significantly different in the EVs of the subjects with post-transplant CKD versus the healthy controls. The heat map was created via Morpheus, https://software.broadinstitute.org/morpheus. *ABV:* CKD: chronic kidney disease, Transplant: post-transplant CKD, CDK8/CCNC: cyclin C, GCKR: glucokinase regulator, MAPK: mitogen-activated protein kinase, CA: carbonic anhydrase, FUT: fucosyltransferase, CCL15: Chemokine Ligand 15, CXCL16: Chemokine (C-X-C motif) ligand 16, CCL14: C–C Motif Chemokine Ligand 14, MAP2K: Mitogen-activated protein kinase kinase, ERP29: endoplasmic reticulum protein 29, IL-17 RC: interleukin 17 receptor C, CCDC80: coiled-coil domain containing 80, ACP5: acid phosphatase 5, TIMP-1: tissue inhibitor of metalloproteinases, MIA: melanoma inhibitory activity, SLP1: secretory leukocyte protease inhibitor, COL18: collagen 18, FSTL: Follistatin-related protein, CD55/DAF: complement decay-accelerating factor, IGFBP: insulin-like growth factor binding protein, UNC5C: Unc-5 Netrin Receptor C, CTSH: cathepsin H, CFD: complement factor D, TNFRSF1B: tumor necrosis factor receptor superfamily member 1B, TNFRSF21: tumor necrosis factor receptor superfamily member 21, PI: peptidase inhibitor, EPHA: ephrin type-A receptor, TNFRSF1A: tumor necrosis factor receptor superfamily member 1A, PRSS: serine protease, RTN4R: Reticulon 4 Receptor, TSPB2: tryptase beta-2, RARRES-2: retinoic acid receptor responder protein 2, REN: renin precursor, IL12B IL23A: reactome with interleukin-12B and interleukin-23A, SPOCK2: osteonectin, PPIB: Peptidylprolyl Isomerase B, IGF-1R: insulin-like growth factor-1 receptor, PDE7A: Phosphodiesterase 7A, MMP: matrix metalloproteinase, TNFRSF15: tumor necrosis factor receptor superfamily member 15, PSMA: Proteasome Subunit Alpha, STX1A: Shiga toxin type 1 A, TACSTD: Tumor Associated Calcium Signal Transducer, CCL28: C–C motif chemokine 28, EIF5: Eukaryotic Translation Initiation Factor 5, ADGRG5:Adhesion G Protein-Coupled Receptor G5, HAPLN1: hyaluronan and proteoglycan link protein 1, BCL2L1: Bcl-2-like 1, FLT: fms-like tyrosine kinase, HAVCR: Hepatitis A Virus Cellular Receptor, ESM: endothelial cell specific molecule, PDK: phosphoinositide-dependent protein kinase, CCL4L1: C–C Motif Chemokine Ligand 4 Like 1, ADIPOQ: Adiponectin, C1Q And Collagen Domain Containing, ESAM: endothelial cell-selective adhesion molecule, CGA FSH-β: chorionic gonadotropin follicle-stimulating hormone-β, HS6ST: heparan sulfate 6-O-sulfotransferase, DCTPP1: dCTP pyrophosphatase-1, PLAUR: plasminogen activator, urokinase receptor, CGA CGB: chromogranin A and chromogranin B, RBM: RNA-binding protein, ARPP19: cAMP-regulated phosphoprotein-19, CSF: colony stimulating factor, CFB: complement factor B, KIT: tyrosine-protein kinase kit, MET: tyrosine-protein kinase Met, NME: Nucleoside Diphosphate Kinase, ERBB3: erythroblastosis oncogene B-3, KIR3DL2: Killer Cell Immunoglobulin Like Receptor, Three Ig Domains And Long Cytoplasmic Tail 2.

Proteins known to be increased in patients with CKD were found to be elevated in the EV fraction of the CKD patients as compared to the healthy controls, including cystatin C and β2-microglobulin. In addition, the renin precursor protein (REN), which originates in the kidney, was significantly higher in patients with CKD compared to healthy controls. Figure 4a shows the heat map of the EV proteins that differed between the CKD and healthy control groups. The following canonical pathways were significantly upregulated in the EVs of the CKD group versus the healthy controls: T helper cell differentiation, hepatic fibrosis/stellate cell activation, role of macrophages, fibroblasts, and endothelial cells in rheumatoid arthritis, type 1 diabetes signaling, and dendritic cell maturation. The following were the top causal networks identified: TNF-α, leptin, interleukin (IL)-12B, IL-4, and apolipoprotein-E. As shown in Table 4, the following proteins were significantly higher in the EVs of CKD patients versus healthy controls, including: complement factor D (CFD), IGFBP-6, serine protease (PRSS) 2 and 1, and tumor necrosis factor receptor superfamily members (TNFRSF)-1B.

**Table 4:** Proteins identified to be significantly different for EVs of CKD versus EVs of healthy control.

Target	CKD patients relative to healthy		Biological pathways
	CKD/healthy fold change	*p* value	
CFD	2.3	< 0.0001	Immune regulation
REN	2.2	< 0.0001	Hemodynamic regulation
β2-microglobulin	2.0	0.0001	Inflammation
PRSS2	2.0	< 0.0001	Immune system, inflammation
Cystatin-C	2.0	< 0.0001	Protein degradation
TNFRSF1B	1.7	0.0001	Apoptosis

The data are shown ratiometrically for relative fluorescence units (RFU) for CKD/healthy control. IGFBP: insulin-like growth factor binding protein, CFD: complement factor D, REN: renin, TNFRSF1B: tumor necrosis factor receptor superfamily member 1B.

### Proteins altered in EVs of post-kidney transplant CKD patients

Figure 4b illustrates all the proteins that were found to be different between the post-transplant CKD patients and the healthy controls. Of note, similar to the native kidney CKD patients, several biomarkers of CKD were found to be higher in the post-transplant CKD patients including cystatin C, β2-microglobulin, and REN although none achieved statistical significance. In comparing the post-transplant CKD EVs to the healthy controls, IPA identified the following canonical pathways to be significantly upregulated: granulocyte adhesion diapedesis, T helper cell differentiation, agranulocyte adhesion diapedesis, hepatic fibrosis/stellate cell activation, and IL-17. The following were the top causal networks identified: IL-27, nuclear factor κ-B, IL-1 receptor associated kinase 4, TNF receptor associated factor (TRAF)-6, and TNF-α. Of note, none of the individual proteins achieved the statistical significance cut-off < 0.0002.

### Overlapping proteins in the circulating EVs of native kidney and post-kidney transplant CKD as compared to the healthy controls

**Figure 5:**
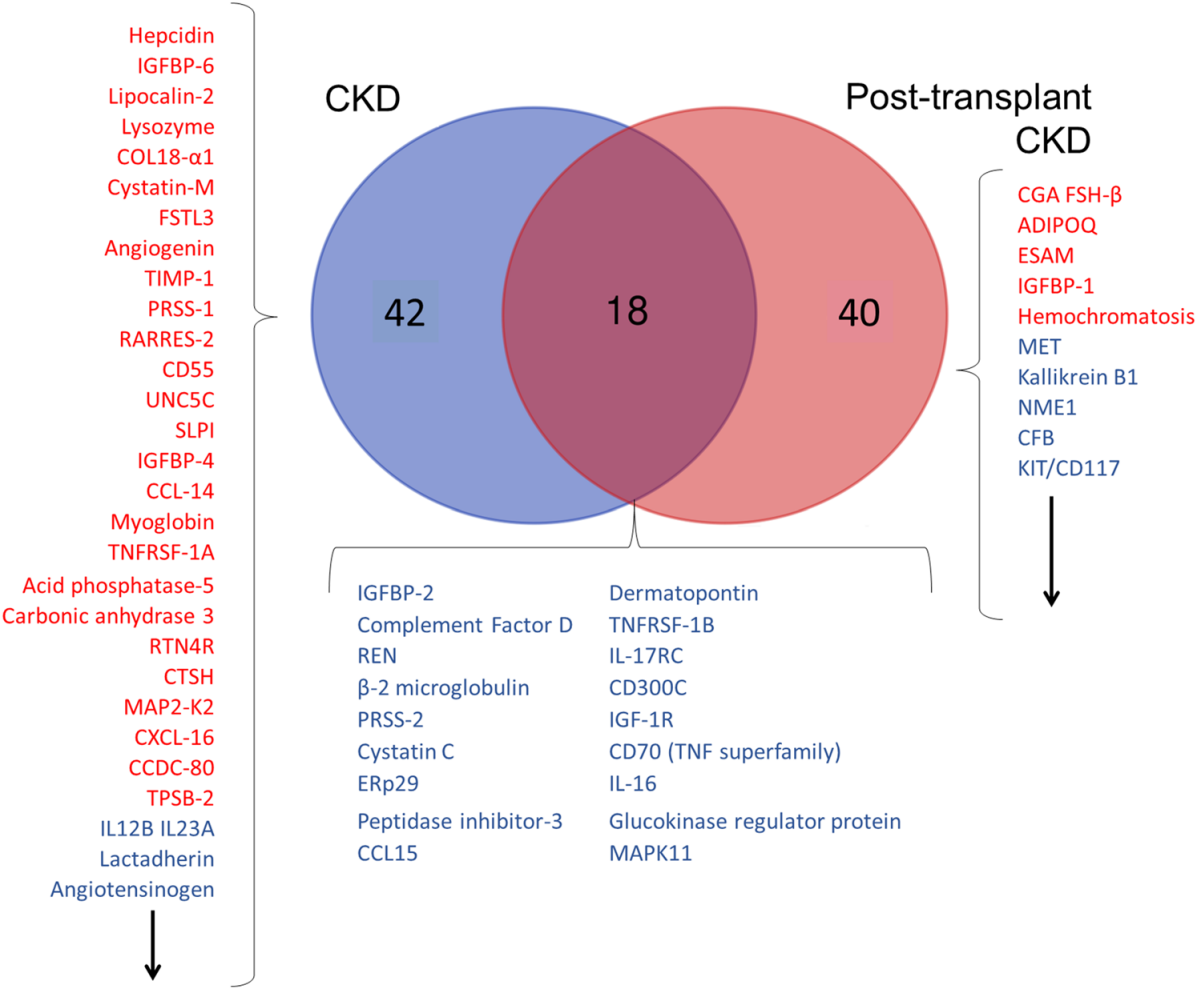
This includes the VENN diagrams for the proteins found to be significantly higher or lower in the EVs of the CKD patients and the post-transplant CKD patients (each group compared to the plasma proteins of the healthy controls). Of those proteins, 18 were found to be shared as they were significantly higher in both the CKD patients and the post-transplant CKD patients. Of note, the red font was used to indicate the proteins that were higher in CKD or in post-transplant CKD (vs. healthy). The blue font was used to denote the proteins that were lower in CKD or post-transplant CKD (vs. healthy controls). The arrows denote that there are additional proteins not shown in the figure. For a complete list of the proteins that differed significantly between CKD and healthy controls or post-transplant CKD and healthy controls, refer to supplemental Tables 2S and 3S, respectively. *ABV:* COL18: collagen 18, TIMP-1: tissue inhibitor of metalloproteinases, PRSS: serine protease, RARRES-2: retinoic acid receptor responder protein 2, UNC5C: Unc-5 Netrin Receptor C, SLP1: secretory leukocyte protease inhibitor, CCL14: C–C Motif Chemokine Ligand 14, TNFRSF1A: tumor necrosis factor receptor superfamily member 1A, RTN4R: Reticulon 4 Receptor, CTSH: cathepsin H, MAP2K: mitogen-activated protein kinase kinase, CXCL16: Chemokine (C-X-C motif) ligand 16, CCDC80: coiled-coil domain containing 80, TSPB2: tryptase beta-2, IL12B IL23A: reactome with interleukin-12B and interleukin-23A, REN: renin precursor, ERp29: endoplasmic reticulum protein 29, CCL15: Chemokine Ligand 15, TNFRSF1B: tumor necrosis factor receptor superfamily member 1B, MAPK: mitogen-activated protein kinase, CGA FSH-β: chorionic gonadotropin follicle-stimulating hormone-β, ADIPOQ: Adiponectin, C1Q And Collagen Domain Containing, ESAM: endothelial cell-selective adhesion molecule, MET: tyrosine-protein kinase Met, NME: Nucleoside Diphosphate Kinase, CFB: complement factor B, KIT: tyrosine-protein kinase kit.

We next evaluated which proteins were differentially expressed in EVs from both CKD patients and the post-transplant CKD patients. As in the plasma analysis, we included all the proteins noted to be significantly different in comparing the CKD patients’ EVs or the post-transplant CKD EVs with the healthy control EVs (i.e. all proteins with ratio of CKD/healthy or post-transplant/healthy > 1 or < 1). Eighteen differentially expressed proteins were found to overlap between the CKD and post-transplant CKD groups including biomarkers of kidney disease (cystatin C, β2-microglobulin, and REN) and biomarkers of inflammation (CFD, IGFBP-2, PRSS 2, Chemokine Ligand 15 (CCL-15), and TNFRSF-1B). These data are illustrated in the VENN diagram in Fig. 5. A complete list of all the proteins noted to be different for either the CKD group or the post-transplant CKD groups as compared to the healthy controls can be found in Supplemental Tables 2S and 3S, respectively.

Of the 18 eV proteins increased in both the native kidney CKD and post-kidney transplant CKD groups, β2-microglobulin and cystatin C were found to correlate significantly with uACR. These data are shown in Table 5.

**Table 5:** Proteins with significant correlations with measures of kidney function.

Variable	CKD- EPI eGFR	uACR
Desmocollin 2_P	− 0.76 < 0.0001	0.60 0.0004
Cystatin C_P	− 0.75 < 0.0001	0.57 0.0008
FABPL_P	− 0.64 < 0.0001	0.57 0.0008
REG4_P	− 0.64 < 0.0001	0.37 0.04
IGFBP-6_P	− 0.54 0.002	0.63 0.0001
CD59_P	− 0.70 < 0.0001	0.47 0.008
Ephrin-A4_P	− 0.67 < 0.0001	0.46 0.009
EFNB2_P	− 0.75 < 0.0001	0.48 0.007
Ephrin-A2_P	− 0.69 < 0.0001	0.52 0.003
SMOC1_P	− 0.72 < 0.0001	0.47 0.008
Ephrin-A5_P	− 0.79 < 0.0001	0.55 0.001
β2-microglobulin_MP	− 0.40 0.015	0.45 0.006
Cystatin C_MP	− 0.29 0.08	0.42 0.01

FABPL: fatty acid-binding protein, liver-type, REG4: regenerating islet-derived protein 4, IGFBP-6: insulin-like growth factor-binding protein-6, Ephrin: Eph family receptor interacting proteins, EFN: ephrin, SMOC1: secreted modular calcium-binding protein 1.

### Validation of the SOMAscan results with MSD

**Figure 6:**
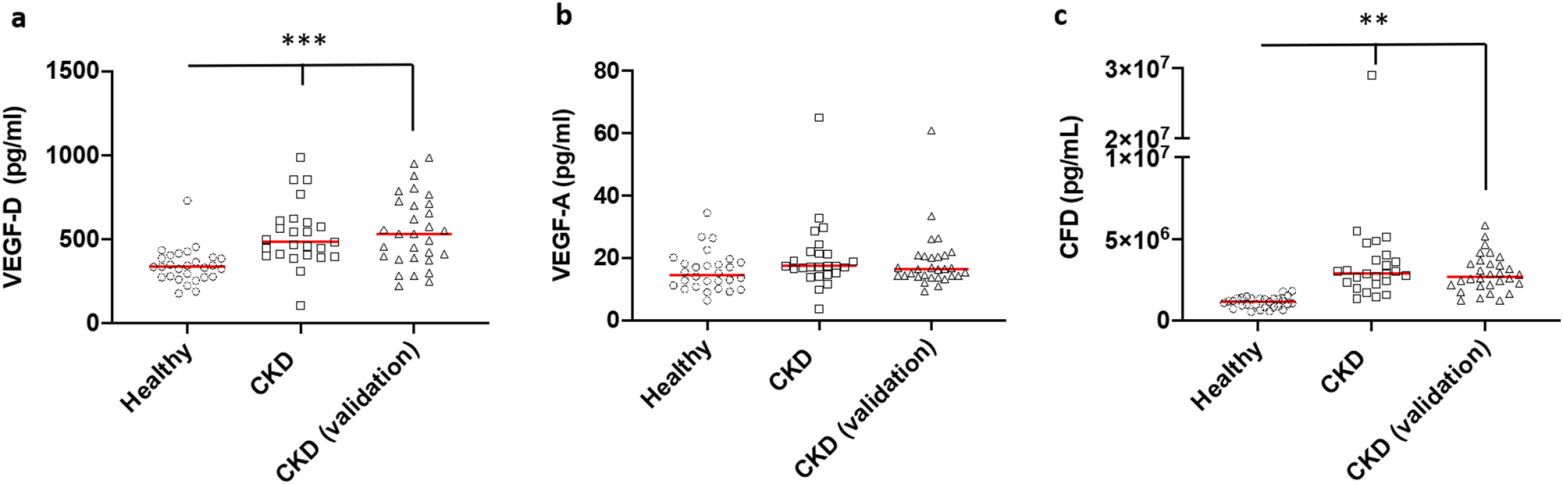
This illustrates the levels of VEGF-D (**a**), VEGF-A (**b**), and CFD (**c**) in the healthy controls and in the subjects with CKD stage 3b and 4 from the first cohort. Additionally, we show the levels of these proteins in the CKD validation cohort. Notably both VEGF-D and CFD were increased in the 2 CKD cohorts as compared to the healthy controls. VEGF: vascular endothelial growth factor, CFD: complement factor D. The figure was created via Prism 9, https://www.graphpad.com/scientific-software/prism/.

Ephrin B2 was measured via ELISA and was detectable in 2 samples in the healthy controls, 6 samples for the CKD samples, and in 11 samples of the validation cohort. Mean(SD) were 9.5(35.9), 14.5(37.1), and 66.4(114.8) pg/mL in the healthy controls, CKD samples, and CKD validation cohort respectively (*p* value = 0.0057). High sensitivity MSD assays are available for CFD, VEGF-A, and VEGF-D, three of the analytes measured by the SOMAscan assay. We therefore used MSD assays to confirm the SOMAscan results for these proteins, and we also validated these findings using samples from a second cohort of CKD patients. VEGF-D and CFD levels were significantly elevated in the CKD samples when measured with MSD, similar to the results obtained by SOMAscan. Similarly, the levels of both proteins were significantly higher in the validation cohort compared to healthy controls. Although VEGF-A levels were higher in the CKD samples than in the healthy control samples by SOMAscan, the levels of VEGF-A measured via MSD in both CKD cohorts were not significantly different from the healthy controls. These data are shown in Fig. 6.

## Discussion

In this study, we sought to identify new biomarkers of inflammation and CVD in patients with CKD by analyzing the proteome of plasma samples using an aptamer-based array in a non-biased manner. We compared samples from patients with native kidney CKD and post-kidney transplant CKD (stages 3 and 4) in order to identify common pathways associated with increased risk of CVD in both patient groups, as the increased risk of CVD persists post kidney transplantation. In addition, the inclusion of patients with post-transplant CKD also allowed us to examine whether markers of inflammation in CKD are affected by treatment with standard immunosuppressive drugs. This approach identified plasma proteins that were differentially and significantly expressed in plasma of patients with native and post-transplant CKD compared to healthy controls. Furthermore, EV proteins were identified in both native kidney CKD and post-transplant CKD. Pathway analysis revealed significant activation of angiogenic and inflammatory pathways in the native and post-transplant CKD as compared to the healthy controls. Many of the identified proteins were significantly correlated with eGFR, and several proteins correlated with albuminuria, which is often used as a marker of microvascular damage. We confirmed the elevations in several analytes including Ephrin B2 ligand (EFN-B2), VEGF-D, and CFD in CKD using ELISA and MSD (a different high sensitivity assay). We also verified that these proteins were elevated in plasma samples from a second cohort of patients with CKD. We had previously described dysregulation of the alternative pathway of complement in this group of subjects^14^. That CFD is elevated in the validation cohort utilizing a different method of detection supports the robustness of this finding.

Examination of the EV proteome revealed that this fraction contained an almost completely different subset of proteins than that seen in whole plasma. By analyzing circulating EVs we were able to identify additional pathways that were differentially activated in CKD patients compared to healthy controls. There was a prominent pro-inflammatory profile in the EV proteome of CKD patients, including markers of T-helper cell activation, complement, and TNF pathways activation in both native and post-transplant CKD groups as compared to the healthy controls. Importantly, several biomarkers of kidney disease known to be produced in excess by injured kidneys were only detected in the EVs, including β2-microglobulin, and the renin precursor protein. These findings suggest that the analyzed circulating EVs, at least partially, originated in the kidney. As such, some of the identified proteins could potentially predict CKD progression. Alternatively, our findings may reflect a pathogenic or protective role in kidney disease progression.

This analysis identified several novel biomarkers of angiogenesis in patients with CKD. In adult organisms, angiogenesis, the formation of blood vessels from preexisting vasculature, is an adaptive mechanism in response to hypoxia and vascular injury^38^ that culminates in the activation of pro-angiogenic growth factors (such as VEGFs and angiopoietins) and their receptors. Vascular injury is also associated with increased vascular permeability, migration of endothelial cells to the site of injury, extracellular matrix remodeling, and ultimately the budding of new vessels^39,40^. Several proteins were detected systemically, that signal activated angiogenesis in CKD patients (both native kidney and post-transplant), the most prominent of which belong to the ephrin receptor family. The ephrin receptors consist of the largest family of receptor tyrosine kinases^41^; at least 16 members divided into two classes, A (ephrin A1–A10) and B (ephrin B1–B6)^42^. These proteins require cell to cell interaction in order to bind their membrane-associated ligands; the ephrins^43^. There are 5 ephrin A ligands that bind promiscuously to all ten ephrin A receptors and 3 ephrin B ligands (EFN-B) that interact with the ephrin B receptors^42^. To our knowledge, this is the first report of altered levels of ephrin ligands/Eph receptors in CKD patients. Considering the data above, ephrin B2 ligand (EFN-B2) is of particular interest. EFN-B2 is induced in endothelial cells with angiogenic activation including in response to VEGF^44–46^. In adults, EFN-B2 is upregulated at sites of neovascularization, such as in tumors and wounds^47,48^. In addition to promoting capillary network formation and sprouting angiogenesis, EFN-B2 stimulation in endothelial cells is known to promote inflammatory cell adhesion, migration, and chemotaxis^49,50^, effects that may promote atherosclerotic vascular disease.

In addition to hypoxia, there is also evidence to suggest that inflammation induces EFN-B2. Most pertinent to our discussion, the EFN-B2 promoter is responsive to nuclear factor-κB (NF-κB)^51^, a ubiquitous pro-inflammatory transcription factor that plays an important role in atherosclerosis^52^. As such, the increased levels of EFN-B2 may reflect underlying inflammation and atherosclerosis. Consistent with a role in atherosclerosis, EFN-B2 is upregulated in human atherosclerotic plaques and expressed in endothelial cells at sites of arteriolosclerosis in mice^53,54^. In addition, and some data suggest it interacts with EphB2 receptor on monocytes thus contributing to the release of pro-inflammatory cytokines^54^. Other members of the ephrin ligand/receptor family may also play a role in atherosclerosis, as EFN-B1 and ephrin B2 expression is increased in human carotid artery atherosclerotic plaques^53^. It is possible that the higher detectable levels of EFN-B2 are a biomarker of vascular inflammation and atherosclerotic disease in CKD. We identified a significant correlation between many of the plasma and EV pro-angiogenic proteins with uACR, a surrogate marker of endothelial dysfunction, CVD, and mortality in patients with CKD^35^. Considering the known role of the identified pro-angiogenic pathways in CVD and the identified correlation with uACR, we suspect that the proteins identified via this analysis relate to the increased risk of CVD in patients with CKD. Certainly, data from animal models of CKD indicate that experimental uremia associates with dysfunctional angiogenesis^55^. Alternatively, some of the proangiogenic biomarkers detected are known to promote angiogenesis in a manner that may improve tissue perfusion in models of vascular disease. For example, VEGF-D is significantly associated with mortality in patients with CAD^56^. However, gene transfer of human VEGF-D has shown improved cardiac perfusion in animal models and may be of use in cases of refractory angina, stent restenosis, and peripheral vascular disease. Thus, it is plausible that the detected pathways herein may be protective. Longitudinal studies will be needed to determine whether the identified proteins are predictive of CVD. Further experiments will be also required to determine whether the pathways activated in CKD patients are functionally important to the increased risk of CVD and other systemic complications.

It is important to recognize that dysregulated angiogenesis is known to play a role in certain kidney diseases, most notably diabetic kidney disease^57,58^. In addition, several studies have implicated these pathways in other glomerular diseases^59^. VEGF-A, for example, is highly expressed in the podocyte and has been shown to play an important role in the formation and preservation of a functional filtration barrier. Additionally, the dysregulation of VEGF-A results in glomerular disease characterized by proteinuria^60^. Of note, while VEGF-A was noted to be increased in CKD subjects versus healthy controls, we were unable to reproduce this in the validation cohort. This may be due to higher sensitivity of the originally utilized assay (SOMAscan). EFN-B2 reverse signaling has been shown in response to fibrotic kidney injury and is believed to play a protective role against capillary rarefaction and fibrosis^61^. Not surprisingly, our findings indicated significant activation of the TGF-β pathway. This was common to both groups of CKD patients, native and post-transplant. Chronic induction of TGF-β is known to cause extracellular matrix accumulation^62^ with resultant glomerular and tubulointerstitial fibrosis^63–65^ and hence is believed to play an important role in the progression of kidney disease. As it pertains to our findings here, TGF-β induction is reported to occur in response to tissue hypoxia and in association with the angiogenic response^66^. Some have advocated that in the presence of hypoxia the balance between the pro-angiogenic response and the pro-fibrotic response is tipped so that the pro-fibrotic response is more prominent^67^. Hence, the pro-angiogenic profile we have identified may in fact be a reflection of the pathological processes in the diseased kidney. It is unknown whether pro-angiogenic pathways contribute to kidney disease in post-transplant CKD and perhaps this is a valid path of investigation in the future.

Lastly, while the majority of detected proteins in this analysis are proangiogenic, some are actually anti-angiogenic. For example, we noted significantly higher levels of endostatin, a protein that has been shown to inhibit pathological angiogenesis and may be a target of therapy in the treatment of cancer^68^. Endostatin is a fragment of collagen XVIII that is highly expressed in the renal glomeruli and pertitubular capillaries and is formed during extracellular remodeling^69^. Thus, in addition to its anti-angiogenic effects, endostatin may be a biomarker of fibrosis. Indeed, higher levels of plasma endostatin have been linked kidney function decline in patients with type 2 DM^69^.

One limitation of this study is the small number of patients and our data should be considered hypothesis generating. Future studies can focus on the panel protein biomarkers and pathways identified in this study to validate these findings in larger patient cohorts. CKD is also caused by many different underlying systemic diseases. A larger number of samples will be required to determine whether there are protein biomarkers specific to particular causes of CKD. We were also unable to evaluate whether any of these novel biomarkers predict hard outcomes and future studies will need to examine whether these biomarkers predict CVD or CKD progression. The SOMAscan assay measures a large panel of proteins with very high sensitivity and specificity, but currently it is cost prohibitive to perform it as a high throughput assay. Nevertheless, our approach overcomes several barriers that have hampered biomarker studies in CKD. The use of an aptamer-based array prevented high abundance plasma proteins from obscuring the lower abundance biomarkers. It also allowed us to detect circulating EV proteins with high sensitivity, as EV proteins represent only a small fraction of the protein contained in a plasma sample.

In summary, we have used an aptamer-based proteomics array to identify pathways that are activated in patients with CKD. We analyzed plasma samples as well as isolated EVs, and we identified discrete groups of proteins that are altered in each of these sample types. Our findings indicate activated angiogenesis and inflammatory pathways, raising the possibility that these pathways could represent therapeutic targets. Furthermore, some of these proteins correlated with albuminuria, a biomarker of CVD and kidney disease progression. Future work will be needed to confirm these findings larger cohorts of patients, and to determine whether any of these candidate markers predict CVD and/or CKD progression.

## Supplementary Information

Supplementary Information 1.

Supplementary Information 2.

Supplementary Information 3.

Supplementary Information 4.
