# Supplementary figures and images for: Detection of pro angiogenic and inflammatory biomarkers in patients with CKD
Source: Sci Rep. 2021 Apr 22;11:8786. doi: 10.1038/s41598-021-87710-0 (PMC8062467; doi:10.1038/s41598-021-87710-0)

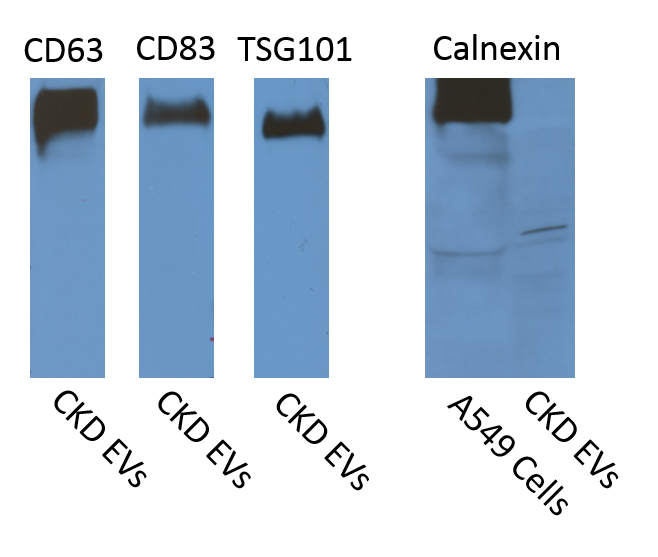

Supplement: Supplementary file 1 — Supplementary Information 1. [file 41598_2021_87710_MOESM1_ESM.tiff]

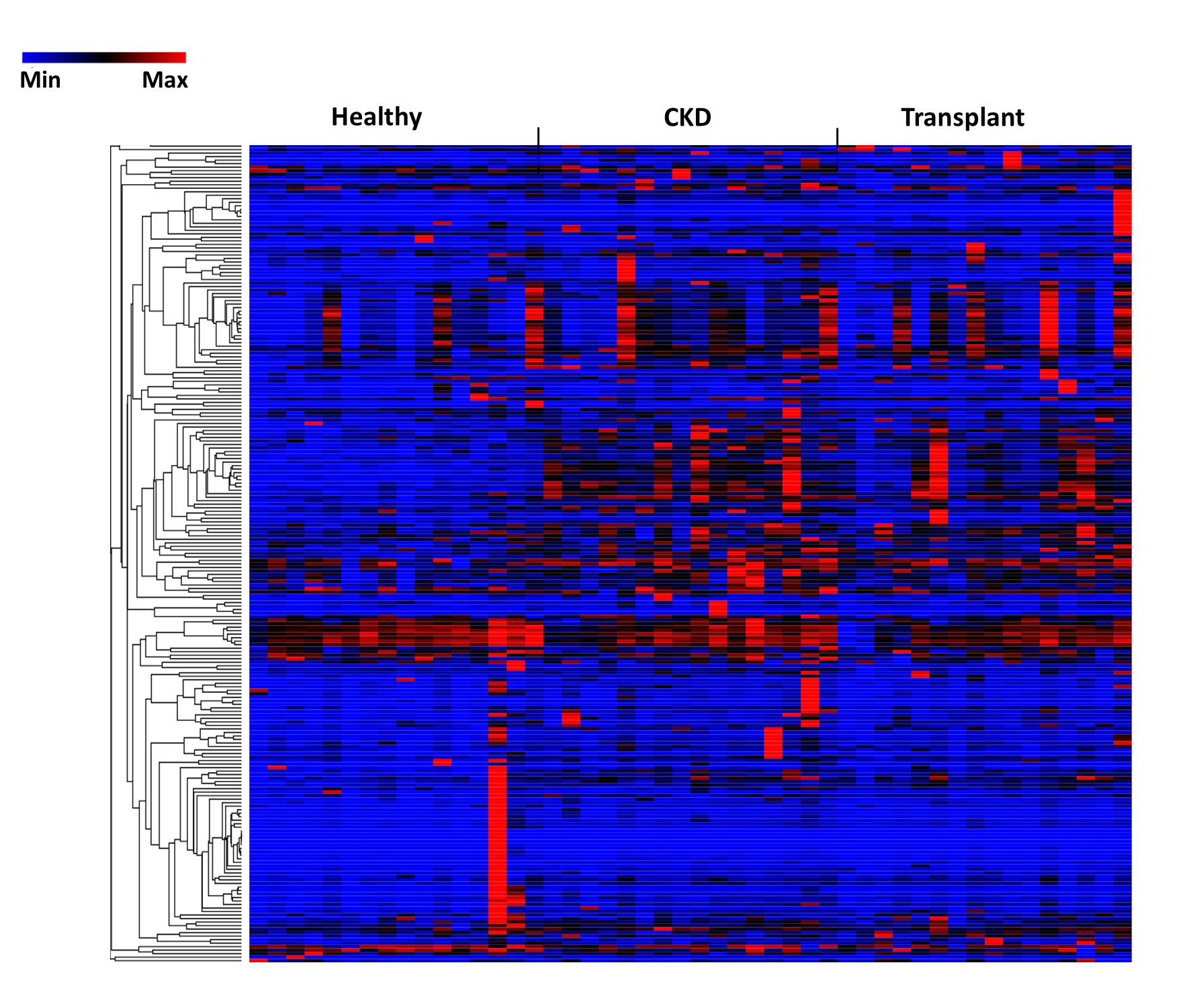

Supplement: Supplementary file 2 — Supplementary Information 2. [file 41598_2021_87710_MOESM2_ESM.tif]

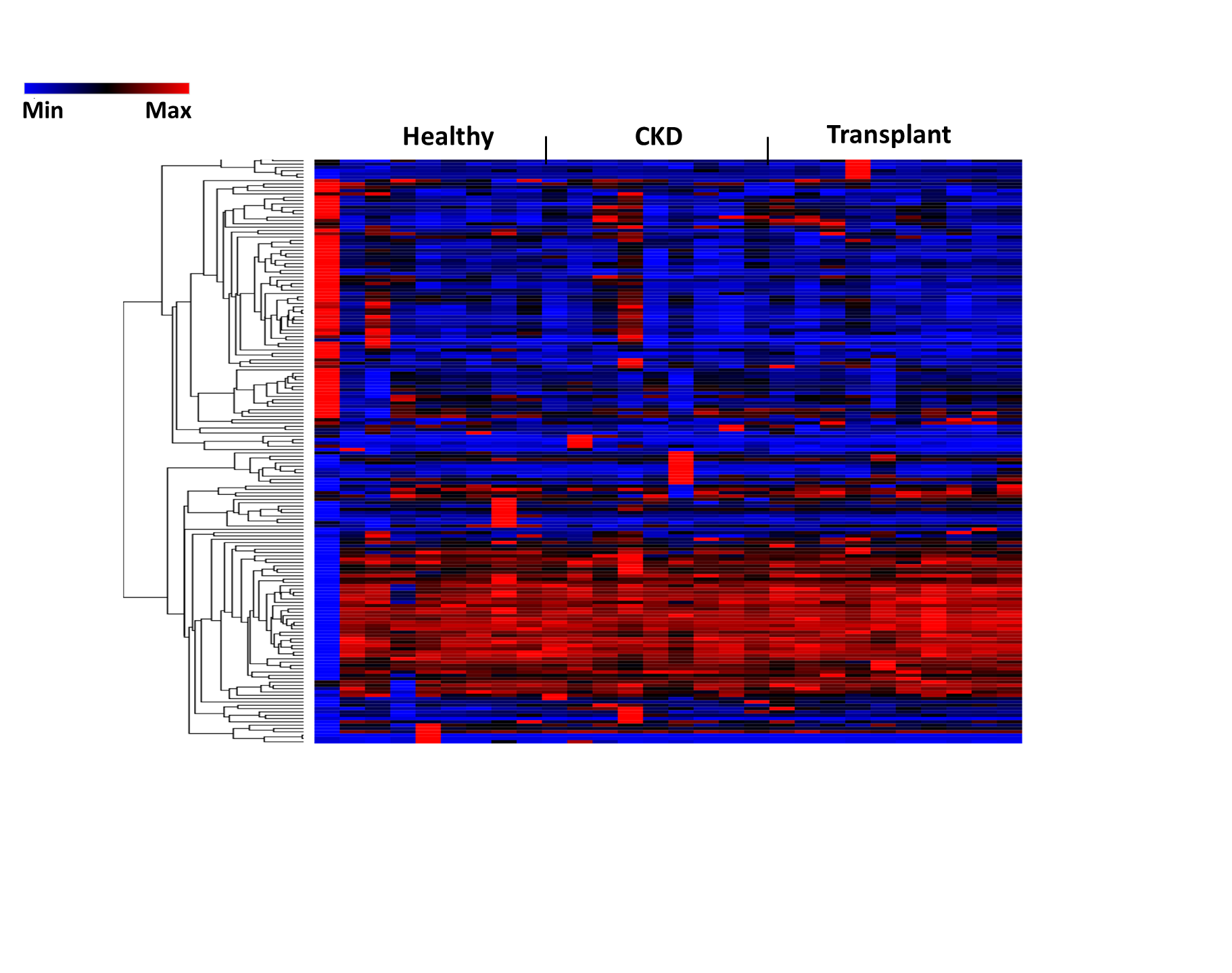

Supplement: Supplementary file 3 — Supplementary Information 3. [file 41598_2021_87710_MOESM3_ESM.tif]
