# Supplementary material for: Detection of pro angiogenic and inflammatory biomarkers in patients with CKD
Source: Sci Rep. 2021 Apr 22;11:8786. doi: 10.1038/s41598-021-87710-0 (PMC8062467; doi:10.1038/s41598-021-87710-0)
Supplement: Supplementary file 4 — Supplementary Information 4. [file 41598_2021_87710_MOESM4_ESM.docx]

**Detection of Pro Angiogenic and Inflammatory Biomarkers in Patients With CKD**

Diana Jalal^1,2*^, Bridget Sanford^3^, Brandon Renner^4^, Patrick Ten Eyck^5^, Jennifer Laskowski^4^, James Cooper^4^, Mingyao Sun^1^, Yousef Zakharia^6^, Douglas Spitz^6,7^, Ayotunde Dokun^1^, Massimo Attanasio^1^, Kenneth Jones^8^, Joshua M. Thurman ^4^

^1^Carver College of Medicine, University of Iowa, Iowa City, IA

^2^Iowa City VA Medical Center, Iowa City, IA

^3^Dept of Pediatrics, University of Colorado Anschutz Medical Center, Aurora, CO, USA

^4^Renal Division, University of Colorado Anschutz Medical Center, Aurora, CO 80045, USA

^5^Institute for Clinical and Translational Science, University of Iowa, Iowa City, IA, USA

^6^Holden Comprehensive Cancer Center, Department of Internal Medicine (Hematology & Oncology), University of Iowa, Iowa City, IA

^7^Free Radical and Radiation Biology Program, University of Iowa, IA

^8^Harold Hamm Diabetes Center, University of Oklahoma School of Medicine, Oklahoma City, OK

**Corresponding author: *** Diana Jalal M.D.

Address: Carver College of Medicine, Department of Internal Medicine, Division of Nephrology

Street Address: 200 Hawkins Dr., E300C-GH, Iowa City, IA, 52242

Email address: diana-jalal@uiowa.edu

Phone: (319) 356-3971

Fax: (319) 356-2999

Supplemental Tables: 3

Supplemental Figures: 3

**Supplemental Table 1S: Clinical characteristics of participants with sufficient sample for extracellular vesicle evaluation**

|  | **CKD**  **Stage 3 & 4**  **(n=16)** | **Post- transplant CKD**  **Stage 3 & 4**  **(n=16)** | **Healthy Control**  **(n=16)** | **P value**  **(ANOVA)** |
| --- | --- | --- | --- | --- |
| **Age (years)** | 59 ± 12 | 48 ± 17 | 36 ± 12 | 0.016 |
| **Gender (female %)** | 44% | 56% | 78% | 0.5 |
| **Race (Caucasian %)** | 78% | 100% | 78% | 0.50 |
| **History of DM (%)** | 47% | 7% | 0% | 0.05 |
| **BMI (kg/m^2^)** | 27.9 ± 3.3 | 24.7 ± 5.7 | 27.3 ± 7.2 | 0.5 |
| **SBP (mmHg)** | 129 ± 12 | 134 ± 9 | 116 ± 10 | 0.004 |
| **DBP (mmHg)** | 81 ± 9 | 89 ± 7 | 70 ± 86.4 | 0.0006 |
| **CKD-EPI eGFR**  **(mL/min/1.73m^2^)** | 40 ± 9 | 50 ± 8 | 99 ± 16 | <0.0001 |
| **ACR (mg/g)** | 3 ± 5 | 2 ± 2 | 0.2 ± 0.1 | 0.5 |
| **BA- FMD (% change)** | 4.6 ± 4.0 | 7.2 ± 4.5 | 9.8 ± 3.1 | 0.03 |

Values are expressed as means ± standard deviation or %= percent of patients; DM= diabetes mellitus; BMI= body mass index; SBP= systolic blood pressure; DBP= diastolic blood pressure; CKD-EPI eGFR= CKD-EPI estimated glomerular filtration rate; ACR= urinary albumin/creatinine ratio; BA- FMD = Brachial Artery Flow-Mediated Dilation (% change).

**Supplemental Table 2S: Proteins identified to be differentially expressed in the extracellular vesicles of CKD subjects (compared to the healthy control subjects)**

| **Target** | **CKD /healthy fold change** | **P-value** |
| --- | --- | --- |
| **Hepcidin** | 2.29 | 0.012 |
| **IGFBP6** | 2.12 | 0.001 |
| **Lipocalin-2** | 1.91 | 0.012 |
| **Lysozyme** | 1.87 | 0.003 |
| **COL18-α1** | 1.86 | 0.004 |
| **Cystatin M** | 1.86 | 0.015 |
| **FSTL3** | 1.75 | 0.001 |
| **Angiogenin** | 1.74 | 0.019 |
| **TIMP1** | 1.71 | 0.032 |
| **PRSS1** | 1.66 | 0.027 |
| **RARRES2** | 1.65 | 0.004 |
| **CD55** | 1.59 | 0.006 |
| **UNC5C** | 1.59 | 0.005 |
| **TNFRSF21** | 1.52 | 0.004 |
| **SLPI** | 1.5 | 0.035 |
| **IGFBP4** | 1.47 | 0.025 |
| **CCL14** | 1.42 | 0.023 |
| **Myoglobin** | 1.4 | 0.002 |
| **TNFRSF1A** | 1.33 | 0.002 |
| **ACP5** | 1.31 | 0.048 |
| **Carbonic anhydrase 3** | 1.3 | 0.022 |
| **RTN4R** | 1.27 | 0.015 |
| **CTSH** | 1.26 | 0.001 |
| **MAP2K2** | 1.26 | 0.024 |
| **CXCL16** | 1.24 | 0.044 |
| **CCDC80** | 1.22 | 0.029 |
| **TPSB2** | 1.2 | 0.035 |
| **FUT5** | 1.16 | 0.012 |
| **EPHA1** | 1.15 | 0.002 |
| **IGF1R** | 1.09 | 0.041 |
| **MMP14** | 1.07 | 0.028 |
| **PDE7A** | 1.07 | 0.021 |
| **TNFSF15** | 1.07 | 0.042 |
| **CDK8 CCNC** | 1.04 | 0.015 |
| **STX1A** | -1.03 | 0.021 |
| **TACSTD2** | -1.04 | 0.033 |
| **PPIB** | -1.05 | 0.027 |
| **PSMA1** | -1.07 | 0.049 |
| **SPOCK2** | -1.09 | 0.003 |
| **IL12B IL23A** | -1.21 | 0.023 |
| **Lactadherin** | -1.41 | 0.037 |
| **Angiotensinogen** | -1.64 | 0.027 |

CKD: chronic kidney disease, IGFBP: insulin-like growth factor binding protein, COL18: collagen 18, FSTL: Follistatin-related protein, TIMP-1: tissue inhibitor of metalloproteinases, PRSS: serine protease, RARRES-2: retinoic acid receptor responder protein 2, CD55/DAF: complement decay-accelerating factor, UNC5C: Unc-5 Netrin Receptor C, TNFRSF21: tumor necrosis factor receptor superfamily member 21, SLP1: secretory leukocyte protease inhibitor, CCL14: C-C motif chemokine 14, TNFRSF1A: tumor necrosis factor receptor superfamily member 1A, , ACP5: acid phosphatase 5, RTN4R: Reticulon 4 Receptor, CTSH: cathepsin H, MAP2K: mitogen-activated protein kinase kinase, CXCL16: Chemokine (C-X-C motif) ligand 16, CCDC80: coiled-coil domain containing 80, TSPB2: tryptase beta-2, FUT: fucosyltransferase, EPHA: ephrin type-A receptor, IGF-1R: insulin-like growth factor-1 receptor, MMP: matrix metalloproteinase, PDE7A: Phosphodiesterase 7A, TNFRSF15: tumor necrosis factor receptor superfamily member 15, CDK8/CCNC: cyclin C, STX1A: Shiga toxin type 1 A, TACSTD: Tumor Associated Calcium Signal Transducer, PPIB: Peptidylprolyl Isomerase B, PSMA: Proteasome Subunit Alpha, SPOCK2: osteonectin, IL12B IL23A: reactome with interleukin-12B and interleukin-23A

**Supplemental Table 3S:** **Proteins found to be differentially expressed in the extracellular vesicles of post-transplant CKD subjects (compared to the healthy controls)**

| **Target** | **Post-transplant CKD /healthy fold change** | **P-value** |
| --- | --- | --- |
| **CGA FSH-β** | 2.41 | 0.027 |
| **ADIPOQ** | 1.98 | 0.043 |
| **ESAM** | 1.43 | 0.025 |
| **IGFBP1** | 1.36 | 0.001 |
| **Hemochromatosis** | 1.23 | 0.033 |
| **ESM1** | 1.19 | 0.030 |
| **IL17F** | 1.19 | 0.020 |
| **PDK1** | 1.17 | 0.024 |
| **CCL4L1** | 1.15 | 0.044 |
| **HS6ST1** | 1.15 | 0.013 |
| **MMP7** | 1.15 | 0.011 |
| **IL13RA1** | 1.12 | 0.032 |
| **Neurexin** | 1.12 | 0.047 |
| **RBM39** | 1.12 | 0.014 |
| **Prefoldin 5** | 1.11 | 0.019 |
| **DCTPP1** | 1.1 | 0.012 |
| **FLT3** | 1.1 | 0.045 |
| **HAVCR2** | 1.1 | 0.035 |
| **BCL2L1** | 1.09 | 0.040 |
| **EIF5** | 1.09 | 0.008 |
| **EPHA2** | 1.09 | 0.002 |
| **MAP2K1** | 1.09 | 0.035 |
| **IL20** | 1.08 | 0.009 |
| **TYRO3** | 1.08 | 0.026 |
| **ADGRG5** | 1.07 | 0.022 |
| **CD48** | 1.07 | 0.002 |
| **CGA CGB** | 1.07 | 0.048 |
| **PLAUR** | 1.07 | 0.033 |
| **EPHA5** | 1.06 | 0.028 |
| **HAPLN1** | 1.06 | 0.025 |
| **CCL28** | 1.05 | 0.038 |
| **KIR3DL2** | -1.05 | 0.048 |
| **ARPP19** | -1.11 | 0.000 |
| **ERBB3** | -1.12 | 0.031 |
| **CSF1** | -1.14 | 0.035 |
| **MET** | -1.41 | 0.027 |
| **Kallikrein B1** | -1.48 | 0.039 |
| **NME1** | -1.54 | 0.033 |
| **CFB** | -1.59 | 0.008 |
| **KIT** | -1.63 | 0.002 |

CGA FSH-β: chorionic gonadotropin follicle-stimulating hormone-β, ADIPOQ: Adiponectin, C1Q And Collagen Domain Containing, ESAM: endothelial cell-selective adhesion molecule, IGFBP: insulin-like growth factor binding protein, ESM: endothelial cell specific molecule, IL: interleukin, PDK: phosphoinositide-dependent protein kinase, CCL4L1: C-C Motif Chemokine Ligand 4 Like 1, HS6ST: heparan sulfate 6-O-sulfotransferase, MMP: matrix metalloproteinase, IL-13Ra1: interleukin 13 Receptor Subunit α 1, RBM: RNA-binding protein, DCTPP1: dCTP pyrophosphatase-1, FLT: fms-like tyrosine kinase, HAVCR: Hepatitis A Virus Cellular Receptor, BCL2L1: Bcl-2-like 1, EIF5: Eukaryotic Translation Initiation Factor 5, EPHA: ephrin type-A receptor, MAP2K: mitogen-activated protein kinase kinase, ADGRG5:Adhesion G Protein-Coupled Receptor G5, CGA CGB: chromogranin A and chromogranin B, PLAUR: plasminogen activator, urokinase receptor, HAPLN1: hyaluronan and proteoglycan link protein 1, CCL28: C-C motif chemokine 28, KIR3DL2: Killer Cell Immunoglobulin Like Receptor, Three Ig Domains And Long Cytoplasmic Tail 2, ARPP19: cAMP-regulated phosphoprotein-19, ERBB3: erythroblastosis oncogene B-3, CSF: colony stimulating factor, MET: tyrosine-protein kinase Met, NME: Nucleoside Diphosphate Kinase, CFB: complement factor B, KIT: tyrosine-protein kinase kit

**Figure legends**

**Supplemental Figure 1S.** Extracellular vesicles (EVs) were isolated from the plasma of a patient with CKD and was used to make a protein lysate. The proteins were separated by electrophoresis and probed on separate blots for CD63, CD83, TSG101, and calnexin. Bands were seen on the blots probed for CD63, CD83, and TSG101. A549 cell lysate was used as a positive control for the antibody to calnexin. Only faint bands were seen in the EV lysate probed for calnexin.

**Supplemental Figure 2S.** **Overview of the plasma proteome in healthy controls and patients with native kidney and post-transplant chronic kidney disease.** Shown is the heat map for the plasma proteomics analysis for the 3 study groups including the healthy controls (Healthy), the patients with stage 3 or 4 CKD (CKD), and the post-transplant patients with stage 3 or 4 CKD (Transplant). Of note, the scale utilizes the blue color to show lower detection levels and progresses to the red color which signifies the highest levels detected.

The heat map was created via Morpheus, <https://software.broadinstitute.org/morpheus>

**Supplemental Figure 3S.** **Overview of the extracellular vesicle proteome in healthy controls and patients with native kidney and post-transplant chronic kidney disease.** This heatmap shows the proteins detected in the extracellular vesicles of the subjects in the 3 study groups including the healthy controls (Healthy), the stage 3 or 4 CKD patients (CKD), and the post-transplant CKD patients with stage 3 or 4 CKD (Transplant). Similar to the plasma data, the scale progresses from the blue color, which denotes lower protein levels, to the red color which denotes higher detectable protein levels.

The heat map was created via Morpheus, <https://software.broadinstitute.org/morpheus>
